# Facile *in-situ* growth of Ag/TiO_2_ nanoparticles on polydopamine modified bamboo with excellent mildew-proofing

**DOI:** 10.1038/s41598-019-53001-y

**Published:** 2019-11-11

**Authors:** Gonggang Liu, Zhou Lu, Xiu Zhu, Xiaoqing Du, Jinbo Hu, Shanshan Chang, Xianjun Li, Yuan Liu

**Affiliations:** 1 Hunan Province Key Laboratory of Materials Surface & Interface Science and Technology, College of Materials Science and Engineering, Central South University of Forestry and Technology, Shaoshan South Road, No. 498, Changsha, 410004 China; 2 School of Materials Science and Energy Engineering, Foshan University, Foshan, 528300 China

**Keywords:** Synthesis and processing, Nanoparticles

## Abstract

Bamboo with the outstanding properties, such as good mechanical strength, fast growth rate and low growth cost, is considered as one of utilitarian structural nature materials. But bamboo is easy to get mildewed resulting in disfiguration and fungi corrosion. In this work, a facile method was developed to improve the mildew-proofing capability of bamboo. Mussel-inspired polydopamine (PDA) with biomimetic adhesion function and highly active functional groups was employed to immobilize highly-dispersed Ag and TiO_2_ nanoparticles on the surface of bamboo via an *in-situ* growth method. Integrating the uniform PDA coating, photocatalytic function of TiO_2_ nanoparticles and bactericidal role of Ag nanoparticles, the mildew-proofing capability of bamboo is enhanced significantly. The results show a non-covalent interaction is more likely to account for the binding mechanism of PDA to bamboo. And the prepared bamboo samples show good photocatalytic performance and have excellent resistance leachability. Meanwhile, the mildew-proofing property of prepared bamboo sample was greatly improved.

## Introduction

Bamboo is considered as an important forest resource and gradually attracts increasing attentions. As a kind of large woody grasses, bamboo has a broad group (1250 species) ranging from 10 cm to 40 m in height and a wide spread application (approximately 1500 commercial applications have been identified) in everyday use by about 2.5 billion people^1^. Due to its good mechanical strength, fast growth rate and low growth cost, bamboo could be used as not only construction material, but a promising raw material for many products instead of the extensive use of steel, concrete and oil byproducts^2–4^. As a construction material, bamboo has the merits of natural and harmonious feeling. And the wide use of bamboo materials can relieve the requirement burden of wood materials from native and planted forests^5^. However, bamboo materials tend to get mildewed because it contains rich sugar, protein, fat and other organic substances^6^, which greatly reduce the use value and range of bamboo products.

Photocatalysis, utilizing solar energy over semiconductors, has recently attracted considerable attentions in the fields of materials science, energy, environmental science, and disinfection^7–12^. Nanosized TiO_2_ with high chemical and thermal stability, non-toxicity, large specific surface area and high catalytic performance has been widely used for various applications^13–17^. And it has been regarded as an effective, economic and environmental way to solve the mildew problem compared with organic anti-mildew agents which are commonly toxic and expensive^18–20^.

However, bare TiO_2_ suffers from low efficiency and narrow light-response range which restrict its application in solar light photocatalysis^21–23^. Furthermore, the agglomeration of TiO_2_ nanoparticles could significantly decrease their photocatalytic activity, and highly-dispersed TiO_2_ nanoparticles are desirable. On the other hand, Ag doping TiO_2_ was adopted to enhance the photocatalytic activity of TiO_2_ under visible light. There is a decrease of recombination of *e*^*-*^/*h*^+^ pairs for Ag doping TiO_2_ because of the formation of a Schottky barrier at the Ag-TiO_2_ interface. Meanwhile the surface Plasmon absorption of Ag could extend its absorption to visible region^24,25^. Besides, silver nanoparticles have excellent germicidal action with bactericidal broad-spectrum characteristic^26^. Hence, TiO_2_ and Ag compounds are also used for effective mould resistance of bamboo^27^. However, high dispersion and immobilization of inorganic nanoparticles on bamboo surface are still challenging for enhancing the mildew-proofing property (sterilizing activity/durability) and saving raw materials. The main reason for the difficulty of immobilizing inorganic nanomaterials on the surface of organic bamboo is the lack of highly active functional groups which could provide strong binding force^28^. Hence, chemicals used for bamboo surface modification are essential for active functional groups.

Inspired by mussel-adhesion phenomena in nature, polydopamine (PDA) possesses outstanding biomimetic adhesion function and highly active functional groups^29^. Here, PDA was used to modify the surface of bamboo in order to uniformly immobilizing nanoparticles. Due to its adhesive ability and functional groups of amino/phenol hydroxyl^30,31^, PDA as an adhesive layer could be easy to anchor on the bamboo surface, and these highly active functional groups make high dispersion and immobilization of inorganic nanoparticles possible. In this paper, the impregnation-adsorption and *in situ* growth methods were used to obtain highly dispersed TiO_2_ and Ag nanoparticles on the PDA modified bamboo surface. And the bamboo with excellent mildew-proofing property was obtained integrating PDA coating, photocatalytic function of TiO_2_ nanoparticles and bactericidal role of Ag nanoparticles. It not only provides a new strategy for bamboo mildew-proofing, but also gives an effective way to immobilize inorganic nanoparticles on surface of biomass matrixes.

## Experiment

### Chemicals and materials

The bamboo used in this study was received from Hunan Taohuajiang Bamboo Science & Technology Co., LTD, in China, which was planed the outer and inner layer. Dopamine (DA), Tris-HCl (NH_2_C(CH_2_OH)_3_**·**HCl), Titanium (IV) oxysulfate solution (TiOSO_4_ solution, 15 wt% in dilute sulfuric acid), Silver nitrate (AgNO_3_), Methylene blue (MB) and Ammonia water solution (25 wt%) were purchased from Sigma-Aldrich and used directly. Cellulose, Xylan (represent hemicellulose) and lignin were purchased from Aladdin and used directly. Deionized (DI) water was used as the solvent.

### PDA Modification and Preparation of Ag/TiO_2_/PDA-Bamboo

The bamboo bundles were dried in an oven at 103 ± 2 ^o^C until reaching a constant weight. The immersion solution (0.4 mg/mL) was prepared by dissolving dopamine in Tris-HCl (10 mM) buffer solution with a pH value of 8.5. Then the bamboo bundles were immersed into the solution and stirred for 24 h at 25 °C, allowing for the deposit of a PDA layer on the surface of the bamboo bundles. The as-obtained bamboo samples were washed with deionized water several times and then dried in an oven at 60 °C, denoted PDA-bamboo. The impregnation-adsorption and *in situ* growth method was used to load TiO_2_ and Ag on the PDA-bamboo. First, PDA-bamboo was immersed into dilute TiOSO_4_ solution (0.01 mol/L) for 2 h, drained and immersed into ammonia water solution (2 wt%) successively. Then it was dried in an oven at 60 °C, denoted TiO_2_/PDA-bamboo. Subsequently, TiO_2_/PDA-bamboo was immersed into water solution of AgNO_3_ (0.001 mol/L) for 2 h. The as-obtained bamboo samples were washed with deionized water several times and then dried in an oven at 60 °C, denoted Ag/TiO_2_/PDA-bamboo. Ag loaded on PDA-bamboo was prepared without TiO_2_ loading process, donated Ag/PDA-bamboo. In order to study the effect of PDA modification on nanoparticles loading process, Ag/TiO_2_ loading bamboo sample without PDA modification was also prepared, denoted Ag/TiO_2_-bamboo. A control sample loading TiO_2_ particles via sol-gel method^32^ was provided, denoted TiO_2_-bamboo.

### The mould test and photocatalytic performance

After infected by the mould, the test-bamboo samples were placed in the transparent cultivate box (20 °C and 65% relative humidity), and a simulated sunlight was provided. The growing speed and the developing state of the mould which cultivated on the bamboo samples were observed, recorded and taken a picture. The photocatalytic activities of bamboo samples were measured by degrading aqueous solutions of MB under a xenon lamp light irradiation as solar simulation^33^. Here, bamboo samples were prepared from bamboo powder and TiO_2_/Ag nanoparticles loading with a same method as bamboo bundles. The photocatalyst (0.2 g) was suspended in an aqueous solution (200 mL) containing 2 mg MB. The 752 N UV-Vis spectrophotometer with a detection wavelength at 664 nm was used to analyze the sampled suspension. The changes in maximum absorption versus irradiation time (C/C_0_ versus t) were obtained which reflected the decrease in the MB concentration. To assess the stability and reusability of the catalyst, 5 cycles were conducted with 80 min in every cycle of photocatalytic reaction. The absorption properties of bamboo samples were also researched, and measurement after 5 minutes is performed. The test procedure is similar to the photocatalytic process.

### Characterization

Morphology of the prepared bamboo samples were observed by scanning electronic microscope (Nova, Nano SEM230, USA), transmission electron microscopy, and their energy dispersive system (EDS) were used to analyze the component of the bamboo samples surface. X-ray diffraction (XRD) was analyzed by using a Japan Rigaku D/MAX-2500 instrument with a Cu Ka radiation and a scanning rate of 5 °C/min. Thermogravimetric analysis (TGA) was carried out by a Mettler Toledo TGA-2 thermal gravimetric analyzer in argon with a heating rate of 10 °C/min, and the bamboo powder samples were used for TGA. Fourier transform infrared (FTIR) spectra were obtained on a Nicolet 6700 spectrometer. X-ray photoelectron spectroscopy (XPS, Thermo ESCALAB 250XI) has been used for the investigation of the surface chemical composition of bamboo samples, and bamboo samples were prepared by cutting bamboo matrix into thin slice (length, width and thickness is 5 × 5 × 3 mm). Ultraviolet visible light spectrophotometer (UV-vis, N5000PC) was used to analysis the non-covalent interactions between bamboo and PDA during PDA modification process. First, the UV-vis absorption spectrum of cellulose, Xylan (represent hemicellulose), lignin and bamboo powder were analyzed, and the samples were dispersed in deionized water under ultrasonic for characterization. Then the UV-vis absorption spectra of DA polymerization with and without lignin at a reaction time of 60 min (pH value is 8.5) were analyzed in order to study the interaction between lignin and formed PDA. A fluorescence spectrophotometer (Hitachi, F4600) and an electrochemical station (Chenhua Instruments, CHI1030B) were used to record the photoluminescence (PL) spectra and photocurrent. The Ag content in the sample was analyzed by using Optima 5300 Inductively Coupled Plasmas Atomic Emissive Spectrometry (ICP-AES), Perkin Elmer.

## Results and Discussion

**Figure 1:**
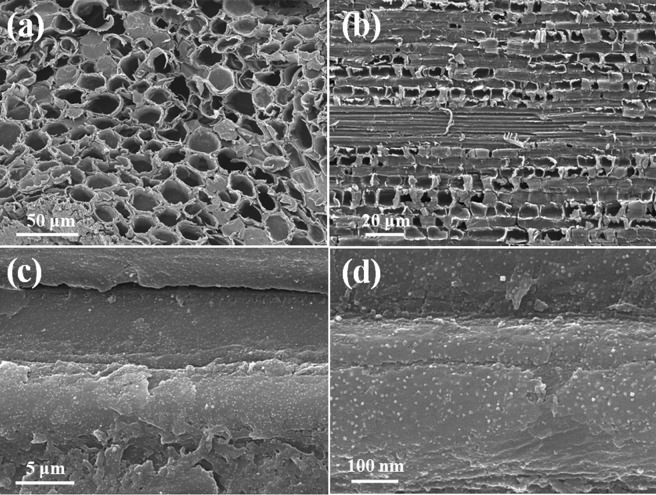
The SEM images of Ag/TiO_2_/PDA-bamboo surface in (**a**) transverse section and (**b**–**d**) longitudinal section.

The microstructure of Ag/TiO_2_/PDA-bamboo surface was characterized by SEM as shown in Fig. 1. Figure 1a,b show the SEM images of bamboo surface in transverse section and longitudinal section, respectively. It shows bamboo has highly developed pore structure and non-uniformly sized pores. As large amounts of nutriment such as starch and sugars exist in these pores^6^, air and moisture could easy enter, then mould tend to have a rapid growth in presence of nutriment in bamboo. Figure 1c,d show the higher magnification images in longitudinal section of bamboo matrix. Plenty of nanoparticles are loaded on the surface of bamboo, and the size of nanoparticles is about a few tens of nanometer. On the other hand, the distribution of nanoparticles on transverse section was also characterized by SEM and elemental mapping as shown in Fig. S1. It shows nanoparticles loading on the walls of micropores could be observed from Fig. S1(a,f). Meanwhile the elemental mapping (Fig. S1b–e) under the magnifications of Fig. S1a is also provided. It can be seen that Ti and Ag elements are homogeneously distributed on the surface of bamboo matrix which indicates nanoparticles are dispersed well, and the result of SEM-EDS spectrum shows nanoparticles contain Ag and Ti elements. In addition, high mass content of Ti (1.27%) and Ag (1.36%) are found in transverse section of Ag/TiO_2_/PDA-bamboo surface. The accurate Ag content in the Ag/TiO_2_/PDA-bamboo matrix was further analyzed by ICP-AES, and Ag loading content is 0.021% which is much lower than that from the result of SEM-EDS spectrum. It indicates Ag nanoparticles were only loading on the surface of bamboo matrix.

**Figure 2:**
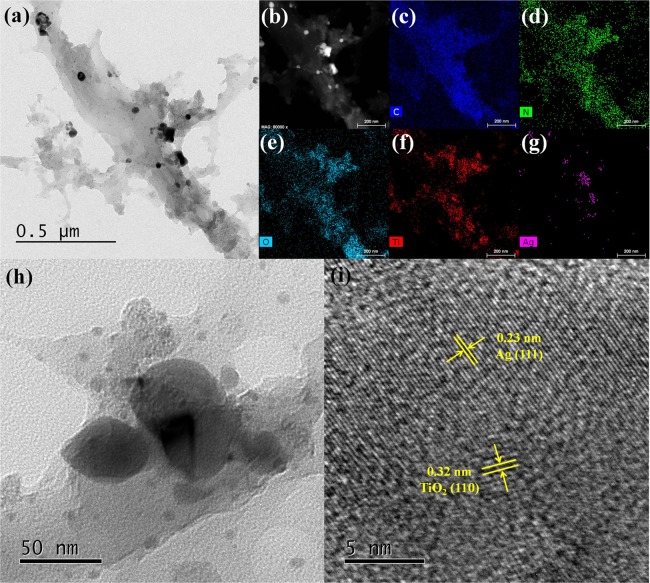
(**a–h**) TEM image of Ag/TiO_2_/PDA-bamboo with corresponding and Ag/TiO_2_ nanoparticles on bamboo, (i) HRTEM image of Ag/TiO_2_ nanoparticles.

Ag/TiO_2_/PDA-bamboo fiber was pulverized and ultrasonic, and its morphology was further characterized by TEM and high-resolution TEM (Fig. 2). From the results of TEM images (Fig. 2a–g), though there are some large agglomerated particles, it can be clearly seen that the Ag and TiO_2_ nanoparticles were homogeneously deposited on the bamboo surface, which is in agreement to the SEM results. And it shows large agglomerated particles contain Ag and Ti elements from TEM-EDS spectrum in Fig. S2. The particle size and crystal structure of the loaded Ag and TiO_2_ nanoparticles were as shown in Fig. 2h,i. Note that the size of small nanoparticles dispersed on bamboo surface is about 15 nm. Besides, Ag with the lattice spacing of 0.23 nm (111) and TiO_2_ with the lattice spacing of 0.32 nm (110) could be found in Fig. 2i, which confirms the identity of the metallic Ag and rutile phase TiO_2_ nanoparticles^21^.

**Figure 3:**
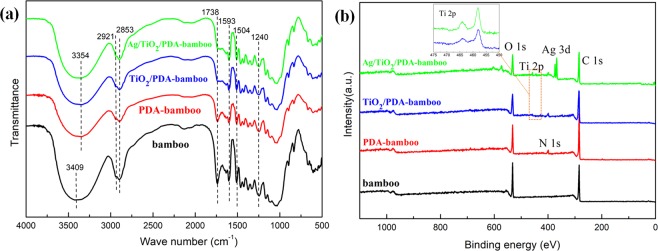
(**a**) FTIR spectra and (**b**) XPS spectra of the control bamboo, PDA-bamboo, TiO_2_/PDA-bamboo, and Ag/TiO_2_/PDA-bamboo samples.

The FTIR spectra of bamboo, PDA-bamboo, TiO_2_/PDA-bamboo, and Ag/TiO_2_/PDA-bamboo samples are as shown in Fig. 3a. For the control bamboo, there is abroad peak at 3409 cm^−1^ assigned to -OH groups on the bamboo, and the band at 2902 cm^−1^ was assigned to C-H stretching vibrations^34^. The peak at 1738, 1593, 1504, 1240 cm^−1^ were assigned to the C = O stretching of the acetyl groups, the aromatic skeletal vibration of lignin, and C-O stretching of the guaiacyl ring, respectively^35^. For the PDA modified bamboo, the peak at 3409 cm^−1^ for the OH groups shifted to 3354 cm^−1^ due to the hydrogen bond formation between PDA and bamboo hydroxyl groups^31^. And the peaks intensity at 1738, 1593, 1504, 1240 cm^−1^ have weakened after PDA modification and nanopartciles loading due to the coverage of PDA coating and nanoparticles. However, the presence of PDA and Ag/TiO_2_ nanoparticles could not be proved by the results from FTIR spectroscopy.

Hence, the surface chemical compositions of bamboo samples were further investigated by X-ray photoelectron spectroscopy, and the results are shown as Fig. 3b. Compared with the XPS survey spectrum of initial bamboo, PDA-bamboo, TiO_2_/PDA-bamboo, and Ag/TiO_2_/PDA-bamboo samples, the peak of N 1 s was observed at 399.4 eV from modified PDA layer, meanwhile Ti and Ag signal appeared after loading Ag/TiO_2_ nanoparticles. The atomic percentages of N, Ti and Ag of Ag/TiO_2_/PDA-bamboo are 4.1%, 1.3% and 2.7% respectively, indicating PDA modification and Ag/TiO_2_ nanoparticles loading. The high-resolution spectra of C 1 s and N 1 s were curve-fitted (Fig. S3a,b). The C 1 s spectra were fitted with three components assigned to C-C(H) at 285.0 eV, C-OH/C-N at 286.4 eV, C = O at 288.0 eV. The N 1 s spectra were fitted with two peaks assigned to amines (-C-NH) at 400.3 eV, aromatic N at 399.5 eV. Aromatic N and amines are from the cyclization reaction occurs during PDA formation and a few open-chain dopamine units on the PDA-bamboo surface, respectively^36^. Besides, the high resolution XPS spectra of the Ti 2p and Ag 3d are shown in Fig. S3c,d. The Ti 2p region can be decomposed into two peaks corresponding to the two typical peaks (Ti 2p_3/2_ and Ti 2p_1/2_) of titanium dioxide. The binding energies of Ti 2p_3/2_ and Ti 2p_1/2_ are observed to be approximately 458.8 and 464.5 eV, and the spin-energy separation is 5.7 eV (Ti^4+^ chemical state) which is the characteristic of TiO_2_^37^. There are two typical peaks of Ag 3d states (Ag 3d_3/2_ and Ag 3d_5/2_) at 374.3 and 368.3 eV, and an energy separation of 6.0 eV between the two peaks are observed which is in agreement with the reference on metallic silver^38^. The results indicate that PDA was successfully coated on the bamboo substrate surfaces and Ag/TiO_2_ particles were deposited on the PDA coated bamboo surfaces.

The crystallographic structures of bamboo (a), TiO_2_/PDA-bamboo (b), and Ag/TiO_2_/PDA-bamboo samples (c) were examined by XRD. As shown in Fig. S4a, there is a typical characteristic peak of cellulose crystal at 16.5° and 22.5° for initial bamboo^39^. For TiO_2_/PDA-bamboo and Ag/TiO_2_/PDA-bamboo samples, these two characteristic peaks are weakened as the coverage of PDA coating and nanoparticles. For TiO_2_/PDA-bamboo and Ag/TiO_2_/PDA-bamboo samples, it reveals a small peak located at 2θ = 25.3°, which correspond to rutile phase TiO_2_ (JCPDS 21-1276). However, it’s not obvious mainly due to low content of TiO_2_ prepared by impregnation-adsorption and *in situ* growth method. Besides, for Ag/TiO_2_/PDA-bamboo sample, the characteristic peaks of metallic silver (PDF#65-2871) at 38.1° and 44.3°^40^ are observed which is consistent with the results of XPS spectra. Because of reductive amino and phenol hydroxyl of PDA, Ag nanoparticles could be easily obtained by a spontaneous *in situ* growth procedure in which Ag^+^ was first adsorbed on the active sites of PDA modified bamboo surface and then grew up. Thermogravimetry analysis was carried out to estimate the PDA content in modified bamboo samples, and the result is as shown in Fig. S4b. The weight loss of initial bamboo was 84.3 wt%. Nevertheless, the weight loss increased to 85.8 wt% for PDA-bamboo, indicating that about 1.5 wt% of PDA was introduced onto the surface of the bamboo.

In an alkalescent environment, dopamine spontaneously polymerized into PDA, and strongly adhered on virtually all inorganic and organic substrate surfaces^41^. However, the detailed binding mechanisms of dopamine remain not clear, especially for biomass such as bamboo, wood. Recent research has showed that the binding mechanism of PDA has intimate relationship with surface chemical composition of substrate mainly including covalent and non-covalent bonding. For covalent bonding, Michael-addition and Schiff-base reactions exist between dopa-quinone and either primary amines or thiols of substrate^42^. As bamboo mainly consists of cellulose, hemicellulose and lignin without any amines or thiols groups, non-covalent bonding is more likely to exist. UV-vis absorption spectrum was used to analysis non-covalent interactions between bamboo and PDA, and the results are as shown in Fig. S5. For cellulose and hemicelluloses, no obvious absorption peak is observed (Fig. S5a). And for lignin, there is an obvious absorption peak at 279 nm which is a typical peak of lignin due to conjugated system from absorption band of aromatic ring^43^. Meanwhile the UV-vis absorption spectrum of bamboo powder was also provided, there is a typical peak of lignin at 279 nm as lignin is an important component of bamboo. In order to study the interaction between lignin and PDA, the UV-vis absorption spectra of DA polymerization with and without lignin at a reaction time of 60 min were also analyzed. As shown in Fig. S5b, there is an obvious absorption peak at 282 nm due to conjugated system from formed aromatic ring for DA polymerization without lignin at a reaction time of 60 min. And for DA polymerization with lignin, the peak has a red shift to 286 nm resulting from π-π non-covalent bonding between lignin and PDA^44^, which confirms there is a non-covalent interaction during the polymerization process.

PDA could be obtained via the oxidative self-polymerisation of dopamine in an alkalescent environment. The process involves oxidation of a catechol to a benzoquinone and cyclization of the primary amine. Then oligomers are formed from further self-condensation^30^. And these oligomers will be probably deposited on the surface of bamboo, due to π-π non-covalent bonding from phenyl structure between lignin and these oligomers (from results of UV-vis absorption spectra) or the hydrogen bond formation between PDA and bamboo hydroxyl groups (from results of FTIR spectra). Meanwhile, these oligomers further aggregate through π-π stacking to form plate-like aggregates, and finally form PDA layer on the surface of bamboo.

The effect of PDA modification on nanoparticles loading process was also studied. Figure S6 shows the SEM images and the element content from the result of SEM-EDS spectrum of bamboo surface in longitudinal section of initial bamboo, Ag/TiO_2_/PDA-bamboo (with PDA), Ag/TiO_2_-bamboo (without PDA) respectively. The results show that no nanoparticles were observed in the control bamboo (Fig. S6a), and nanoparticles are loading on the surface of Ag/TiO_2_-bamboo and Ag/TiO_2_/PDA-bamboo (Fig. S6b,c). But more nanoparticles could be observed on the surface of Ag/TiO_2_/PDA-bamboo with PDA modification. It indicates PDA coating is very beneficial for nanoparticles loading in an *in situ* growth way. The element contents of various samples from the result of SEM-EDS spectra in Fig. S6d further confirm it.

**Figure 4:**
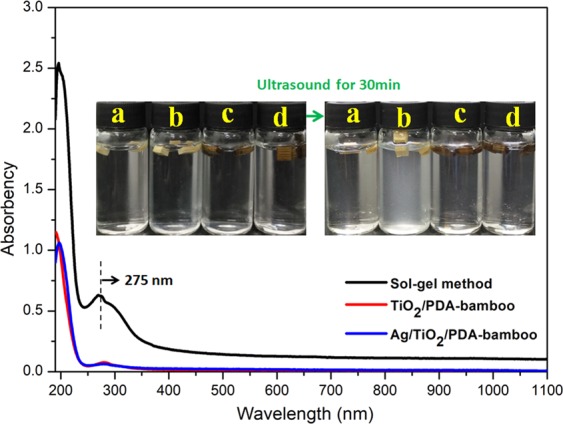
The digital photos of the samples before and after 30 min ultrasound ((**a**) initial bamboo, (**b**) TiO_2_-bamboo, (**c**) TiO_2_/PDA-bamboo, d: Ag/TiO_2_/PDA-bamboo) and UV-vis absorption spectrum of the solutions from the ultrasonic process.

Resistance leachability of nanoparticles loading on bamboo matrix is an important indicator to evaluate the long-term mildew-proofing property and high-risk for eco-toxicity^45,46^. Here, resistance leachability of prepared bamboo samples was studied via an ultrasonic test. In this period, initial bamboo, TiO_2_-bamboo (sol-gel method), TiO_2_/PDA-bamboo, and Ag/TiO_2_/PDA-bamboo samples were treated by ultrasound for 30 min. The digital photos of samples before and after the ultrasound and UV-vis absorption spectra of the solutions from the ultrasonic process were as shown in Fig. 4. The results from digital photos show that only the solution of TiO_2_-bamboo after ultrasound becomes a little turbid. Meanwhile, the results from UV-vis absorption spectrum show there is a very strong absorption for TiO_2_-bamboo due to the absorption of TiO_2_ in UV light region^47^. It indicates that nanoparticles obtained by the *in situ* growth method have less loss compared with traditional sol-gel method. Their SEM images and the element contents from the result of SEM-EDS spectrum were also provided as show in Fig. S7. For the surface of TiO_2_-bamboo, TiO_2_ is morphology of film, and it involves large mass loss of Ti element (about 77%) after ultrasound. However, for TiO_2_/PDA-bamboo and Ag/TiO_2_/PDA-bamboo samples, there is no obvious difference about the nanoparticles distribution and mass loss before and after ultrasound. It indicates *in situ* growth method based on PDA modification owns excellent resistance leachability.

**Figure 5:**
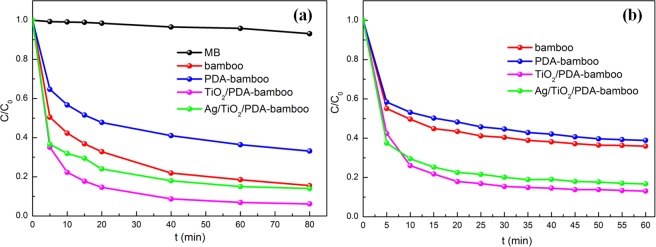
(**a**) Photocatalytic activities of bamboo, PDA-bamboo, TiO_2_/PDA-bamboo and Ag/TiO_2_/PDA-bamboo samples for degradation of MB and (**b**) their absorption properties for MB.

The photocatalytic activities of bamboo, PDA-bamboo, TiO_2_/PDA-bamboo and Ag/TiO_2_/PDA-bamboo samples were studied via MB degradation. The evolutions of MB photodegradation under xenon lamp (solar simulation) are as shown in Fig. 5a. Apparently, only small amount of MB could be photodegraded under xenon lamp without any samples. However, most of MB molecules have been removed for TiO_2_/PDA-bamboo and Ag/TiO_2_/PDA-bamboo samples. Interestingly, for bamboo and PDA-bamboo, high removal rates were also observed with light irradiation, and unmodified bamboo has a higher removal rate than PDA-bamboo. Besides, the effect of pH on the photodegradation of Ag/TiO_2_/PDA-bamboo for MB was also studied, and the corresponding results are shown in Fig. S8. It’s obvious there is gigantic difference between acid and alkaline condition. The results may be due to high adsorption ability of Ag/TiO_2_/PDA-bamboo in an alkaline condition. As solution pH would affect surface binding-sites of the Ag/TiO_2_/PDA-bamboo with abundant oxygenic groups. At lower pH, H^+^ may compete with MB (cationic dye) for the adsorption sites, thereby inhibiting the adsorption of dyes. Otherwise, higher pH is beneficial for MB adsorption resulting from large amounts of exposed adsorption sites^48^. In order to understand the effect of absorption on MB photodegradation, the absorption properties of various bamboo samples were also tested as shown in Fig. 5b. Basically, all samples could reach adsorption equilibrium about 1 h. It is noticeable that all samples have good absorption abilities for MB and the difference of absorption abilities among these bamboo samples are in accordance with their MB removal rate. It indicates the high MB removal rate of prepared bamboo sample in photodegradation process is likely owing to its good absorption ability, and the absorption has more important effects than the photocatalysis on MB removal. The BET results from Fig. S9 show Ag/TiO_2_/PDA-bamboo has a low specific surface area of 11.21 m^2^/g, and the average pore size is 2.86 nm. It illustrates chemical adsorption process from oxygenic groups on surface of Ag/TiO_2_/PDA-bamboo plays a more important role on MB absorption.

In addition, 5 cycles were conducted with 80 min in every cycle of MB photodegradation under xenon lamp, and the results are as shown in Fig. S10. However, after 5-fold continuous decolorization cycles, the MB removal rate decrease a lot mainly due to catalyst contamination result from accumulated MB molecules which are absorbed on Ag/TiO_2_/PDA-bamboo surface. And it could be proved by dark color of the sample with increased cycles and recovery removal rate after light decontamination. For comparison with TiO_2_/PDA-bamboo and Ag/TiO_2_/PDA-bamboo, MB photodegradation ability of Ag/TiO_2_ catalyst^49^ was provided as shown in Fig. S11, and their kinetic linear simulation curves are as shown in Fig. S12. Ag/TiO_2_/PDA-bamboo has lower photodegradation than Ag/TiO_2_ catalyst, but has higher adsorption ability.

To further investigate the mechanism of the photocatalytic activity of Ag/TiO_2_/PDA-bamboo under xenon lamp, the PL spectra and photocurrent were used to understand the separation of photogenerated charges. The results are as shown in Fig. S13. The PL spectra of Ag/TiO_2_/PDA-bamboo and TiO_2_/PDA-bamboo samples were acquired using an excitation with a wavelength of 260 nm in Fig. S13a. The PL spectra of Ag/TiO_2_/PDA-bamboo and TiO_2_/PDA-bamboo samples present an emission peak at about 440 nm. Moreover, the emission intensity of Ag/TiO_2_/PDA-bamboo is lower than that of TiO_2_/PDA-bamboo sample without Ag. A lower PL-peak intensity indicates there is a high separation efficiency of photogenerated charges in presence of Ag which could enhance the photocatalytic activity of catalyst^16^. The transient photocurrent responses of Ag/TiO_2_/PDA-bamboo and TiO_2_/PDA-bamboo sample under visible light are shown in Fig. S13b. TiO_2_/PDA-bamboo sample has no photocurrent response under visible light, and Ag/TiO_2_/PDA-bamboo has an obvious photocurrent response suggesting that the separation efficiency of photogenerated carriers is enhanced by the presence of Ag^50^. Trapping experiments of active species were carried out to confirm the active species that contribute to the photocatalytic degradation MB experiments over Ag/TiO_2_/PDA-bamboo. The hydroxyl radical (•OH), hole (*h*^+^), and superoxide radical anion (•O^2−^) were captured by corresponding trapping agents of Methanol (MeOH), triethanolamine (TEOA), and *p*-benzoquinone (BQ)^17^. As shown in Fig. S14, when MeOH or BQ is added into the reactive system, the photodegradation efficiency decreased sharply which demonstrated that •OH and •O^2−^ are the active species. However, the photodegradation efficiency increased when TEOA is added. It is speculated that the hydrolysis of TEOA changes pH of the solution into alkalinity which enhance MB adsorption ability of Ag/TiO_2_/PDA-bamboo. In a word, the enhanced photocatalytic activity of Ag/TiO_2_/PDA-bamboo under solar light is beneficial to photocatalytic sterilization.

**Figure 6:**
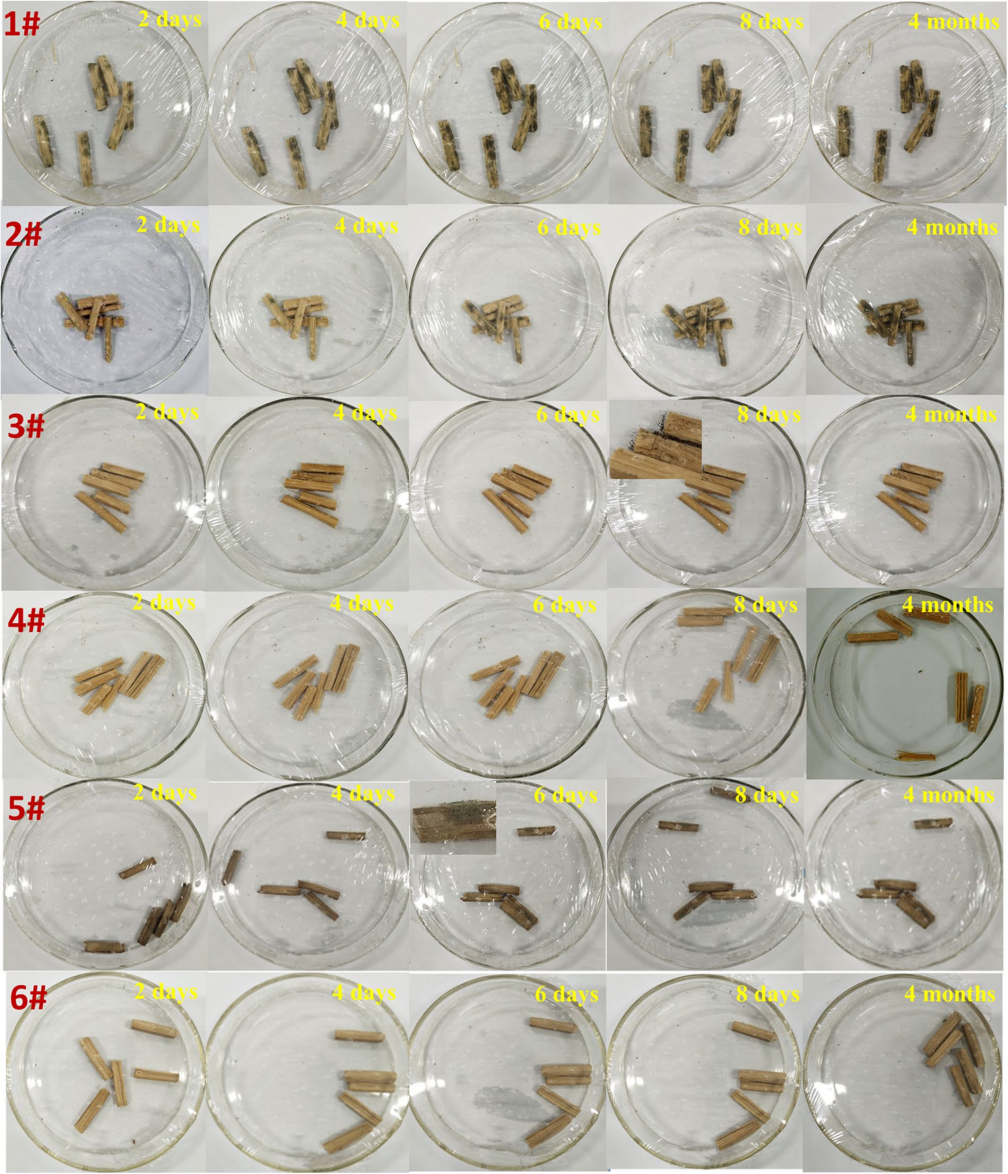
The digital photos of initial bamboo (1#), PDA-bamboo (2#), TiO_2_-bamboo (3#), TiO_2_/PDA-bamboo (4#), Ag/PDA-bamboo (5#) and Ag/TiO_2_/PDA-bamboo (6#) at different time under a constant temperature and high humidity condition.

In order to study the mildew-proofing abilities of prepared bamboo samples, the growing speed and the developing state of the mould which cultivated on the bamboo bundle samples were observed. The digital photos of initial bamboo (1#), PDA-bamboo (2#), TiO_2_-bamboo (3#), TiO_2_/PDA-bamboo (4#), Ag/PDA-bamboo (5#), Ag/TiO_2_/PDA-bamboo (6#) at different time are shown in Fig. 6. It shows that only thimbleful mildew can be seen on 1# sample two days later and there is no obvious mildew in other samples. Four days later, a certain amount of mildew appeared on 2# sample, but a large amount of mildew appeared on 1# sample. The color of bamboo sample changes into black after Ag loading, and only thimbleful mildew can be seen on 5# sample six days later. Eight days later, there is sparse mildew on 3# sample. And the mildew was still not found on 4# and 6# samples. Until four month later, only thimbleful white mildew could be observed on 4# sample. At the same time, the mildew was still not discovered. The results proved that PDA coating on bamboo surface (2#) and Ag loading (5#) could improve mildew-proofing to a certain extent. It is mainly because PDA coating plays a role of mechanical isolation which could slow down external material into bamboo pore, and Ag nanoparticles have good germicidal action to some kind of mould^26^. Meanwhile, bamboo samples with TiO_2_ loading via *in situ* growth (4#) have fine effect of mildew-proofing mainly because of photocatalysis^19^. And Ag/TiO_2_/PDA-bamboo (6#) has the best mildew-proofing ability. Hence we infer the mildew-proofing capability of Ag/TiO_2_/PDA-bamboo could be improved significantly by integrating uniform PDA coating, photocatalytic function of TiO_2_ nanoparticles and bactericidal action of Ag nanoparticles. And the possible mechanism of the improved mildew-proofing capability of Ag/TiO_2_/PDA-bamboo is schematically illustrated in Fig. S15.

## Conclusion

In this work, a facile method is developed to increase mildew-proofing capability of bamboo. Mussel-inspired PDA chemistry was used to prepare stable and uniform coating on bamboo surface with highly active functional groups, then Ag and TiO_2_ nanoparticles could be *in-situ* grown and immobilized onto bamboo surface via these highly active functional groups. The results show that the binding mechanism of PDA on bamboo is mainly due to a non-covalent interaction. And after PDA modification, bamboo was covered with highly-dispersed Ag and TiO_2_ nanoparticles. Meanwhile, the Ag and TiO_2_ nanoparticles could be loading on the bamboo surface firmly via this method. Owning to integrating PDA coating, photocatalytic function of TiO_2_ nanoparticles and bactericidal action of Ag nanoparticles, an indication of improvement in mildew-proofing property of bamboo was observed. The research could not only provide a new strategy for bamboo mildew-proofing, but also give an effective way to immobilize inorganic nanoparticles on bamboo or wood.

## Supplementary information

Supporting Information
