# Supplementary material for: Facile in-situ growth of Ag/TiO2 nanoparticles on polydopamine modified bamboo with excellent mildew-proofing
Source: Sci Rep. 2019 Nov 11;9:16496. doi: 10.1038/s41598-019-53001-y (PMC6848128; doi:10.1038/s41598-019-53001-y)
Supplement: Supplementary file 1 — Supporting Information [file 41598_2019_53001_MOESM1_ESM.docx]

# *Supporting information*

Facile *in-situ* growth of Ag/TiO_2_ nanoparticles on polydopamine modified bamboo with excellent mildew-proofing

Gonggang Liu^1^, Zhou Lu^1^, Xiu Zhu^1^, Xiaoqing Du^2^, Jinbo Hu^1^, Shanshan Chang^1^, Xianjun Li^1^ & Yuan Liu^1^

*^1^Hunan Province Key Laboratory of Materials Surface & Interface Science and Technology,* *College of Materials Science and Engineering,* *Central South University of Forestry and Technology, Shaoshan South Road, No. 498, Changsha 410004, China*

*^2^School of Materials Science and Energy Engineering,* *Foshan University, Foshan 528300, China*

*JinboHu*(🖂)*, E-mail:*[*hjb1999@hotmail.com*](mailto:hjb1999@hotmail.com)*; Shanshan Chang*(🖂)*, E-mail:changelxy@hotmail.com*


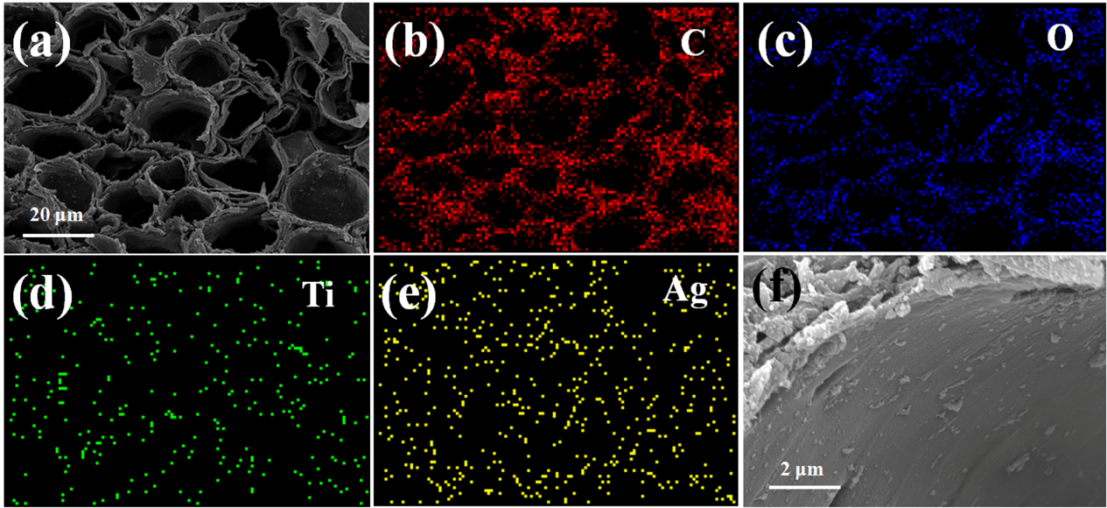


**Fig. S1** The SEM images of Ag/TiO_2_/PDA-bamboo surface in (a) transverse section with (b, c, d, e) C, O, Ti, Ag elemental mapping and (f) larger magnification.


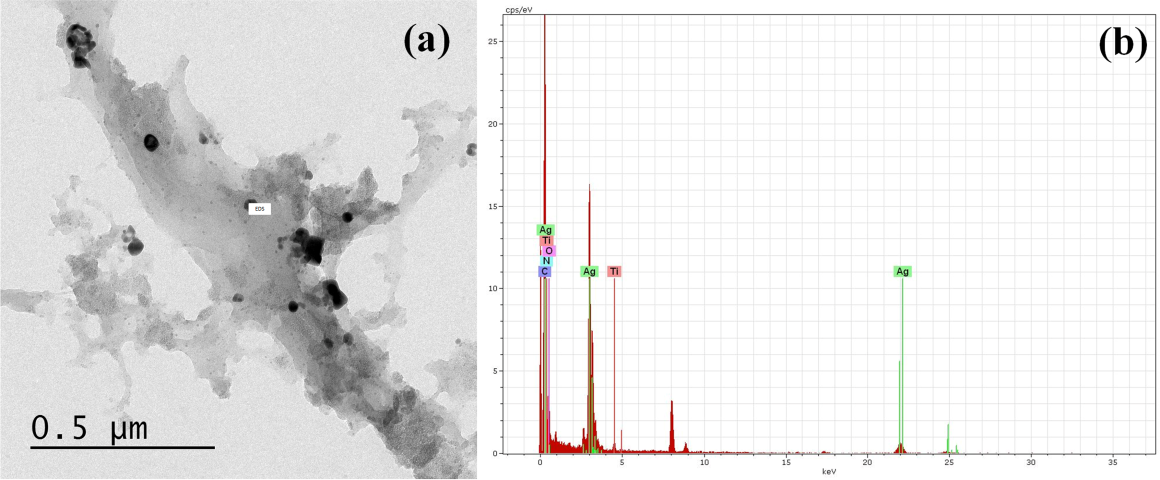


**Fig. S2** TEM image of (a) Ag/TiO_2_/PDA-bamboo and (b) its TEM-EDS spectrum.


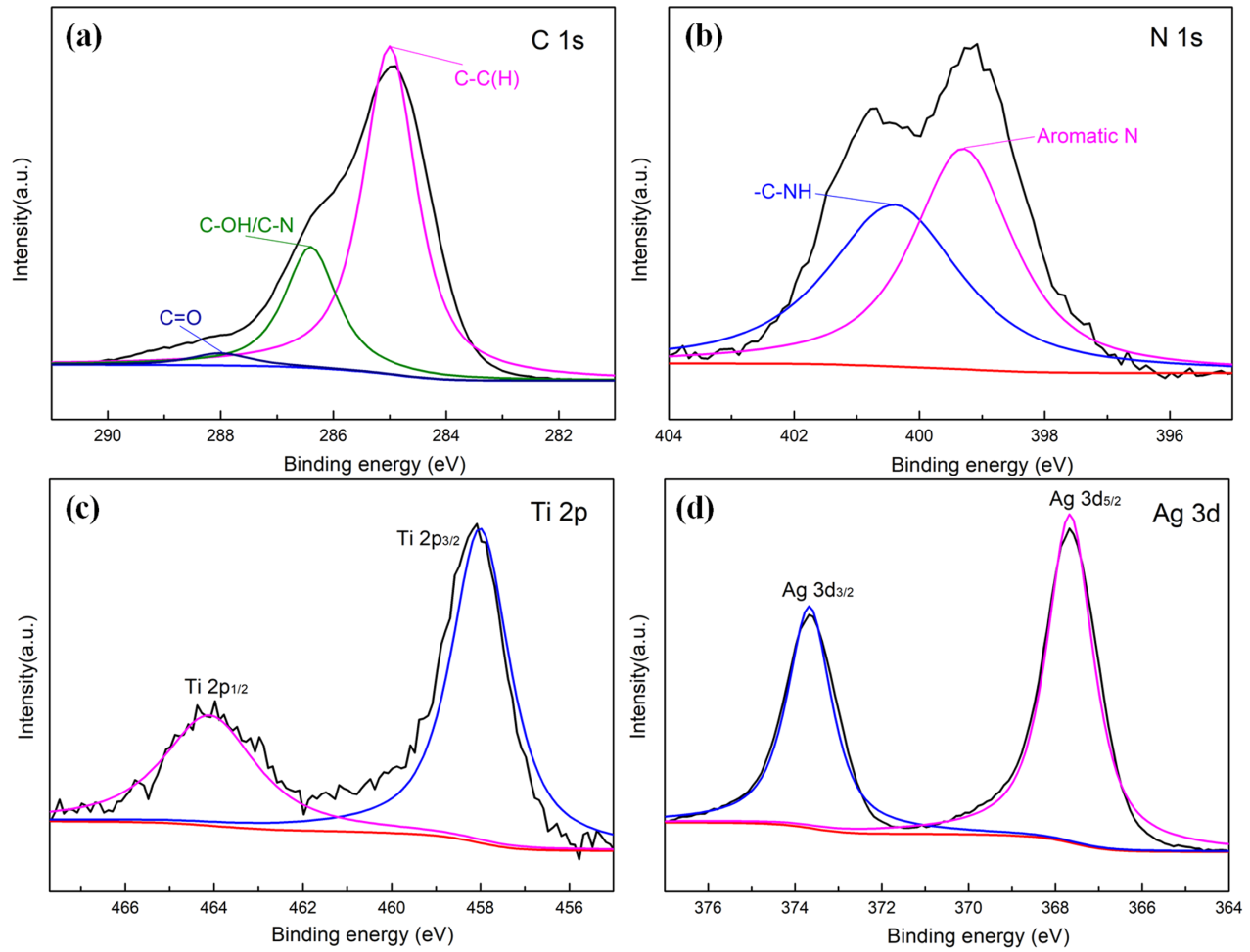


**Fig. S3** The high resolution XPS spectra of (a) the C 1s, (b) N 1s, (c) Ti 2p and (d) Ag 3d of Ag/TiO_2_/PDA-bamboo samples.


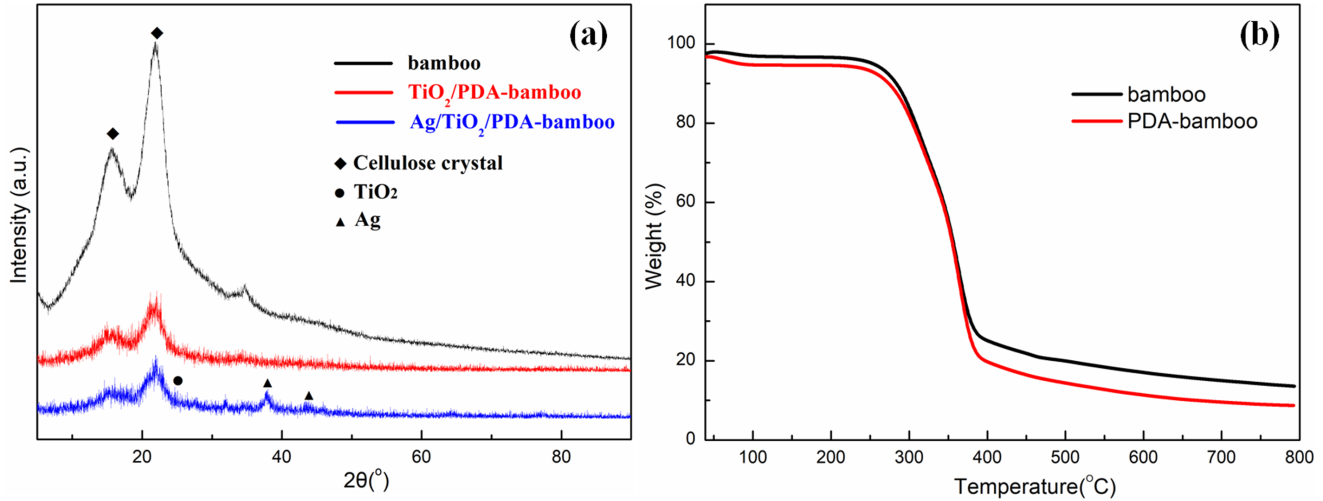


**Fig. S4** (a) XRD spectra of bamboo, TiO_2_/PDA-bamboo, and Ag/TiO_2_/PDA-bamboo samples; (b) TGA curves of bamboo and PDA-bamboo samples.


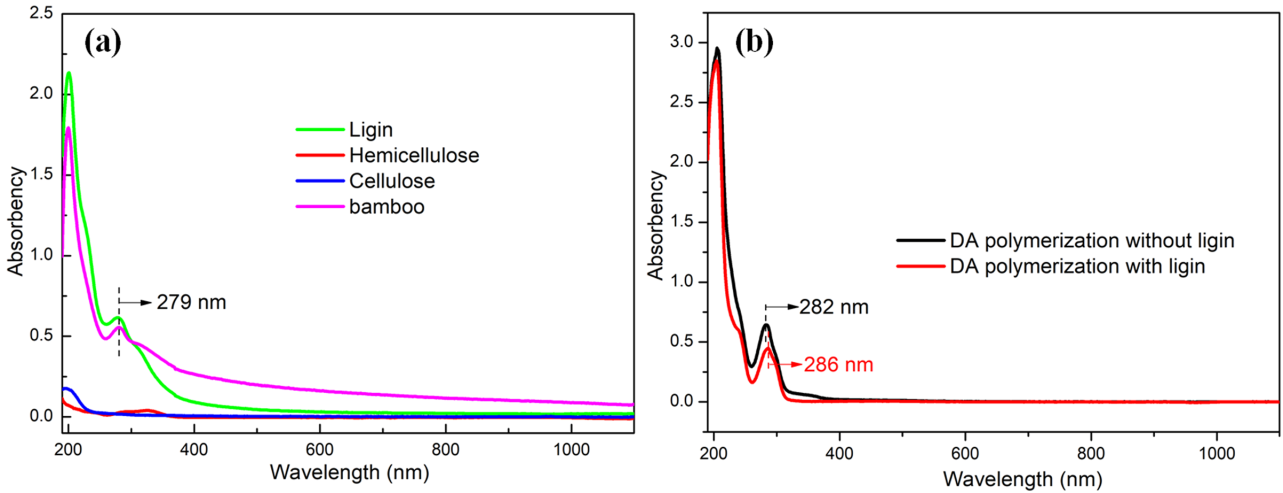


**Fig. S5** UV-vis absorption spectrum of (a) cellulose, hemicellulose and lignin, (b) DA polymerization with and without lignin at a reaction time of 60min.


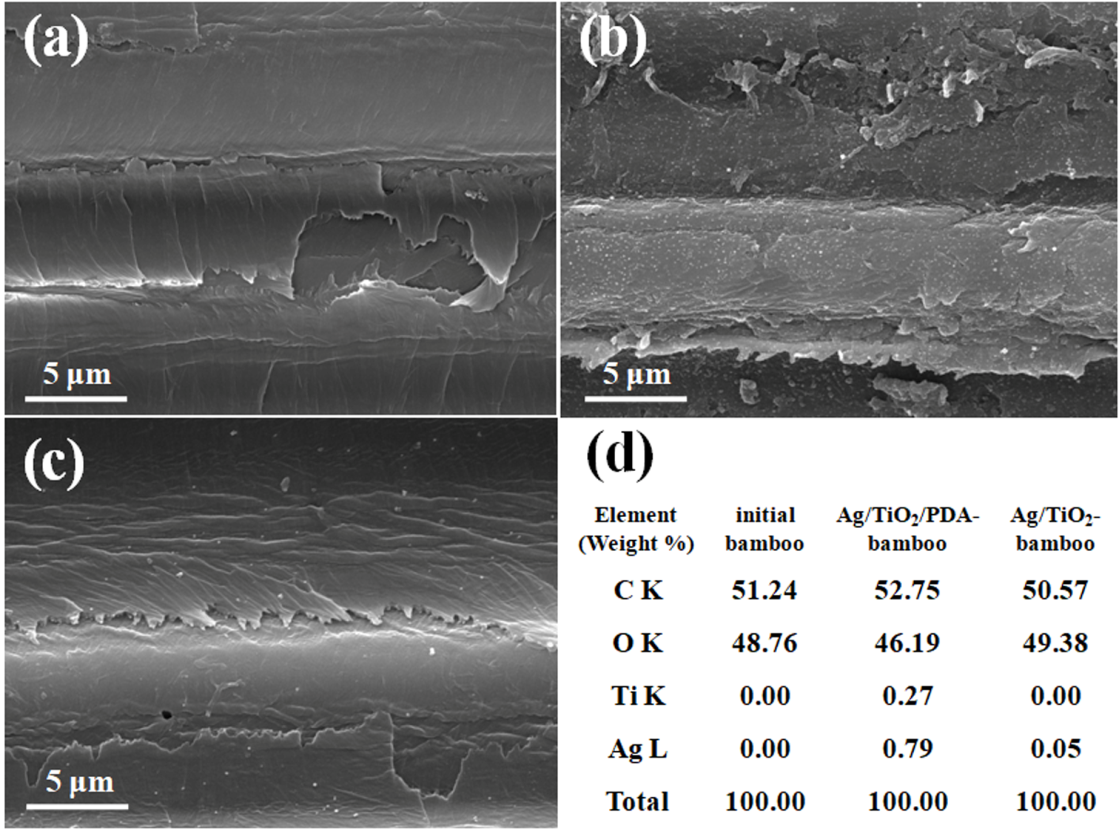


**Fig. S6** The SEM images of various bamboo surface in longitudinal section of (a) initial bamboo, (b) Ag/TiO_2_/PDA-bamboo (with PDA modification) and (c) Ag/TiO_2_-bamboo (without PDA modification), and (d) the element content from the result of SEM-EDS spectrum.


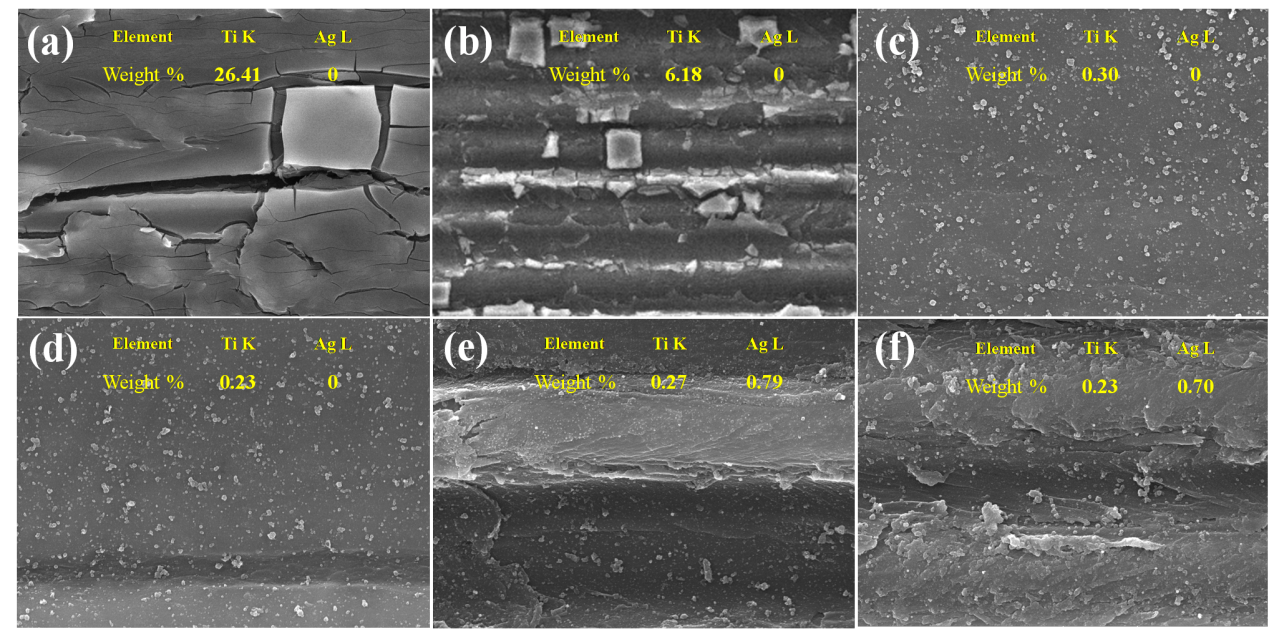


**Fig. S7** The SEM images of (a, b) TiO_2_-bamboo, (c, d) TiO_2_/PDA-bamboo, and (e, f) Ag/TiO_2_/PDA-bamboo samples before and after 30min ultrasound, and the element content from the result of SEM-EDS spectrum.





**Fig. S8** Effect of solution pH on MB degradation of Ag/TiO_2_/PDA-bamboo samples.


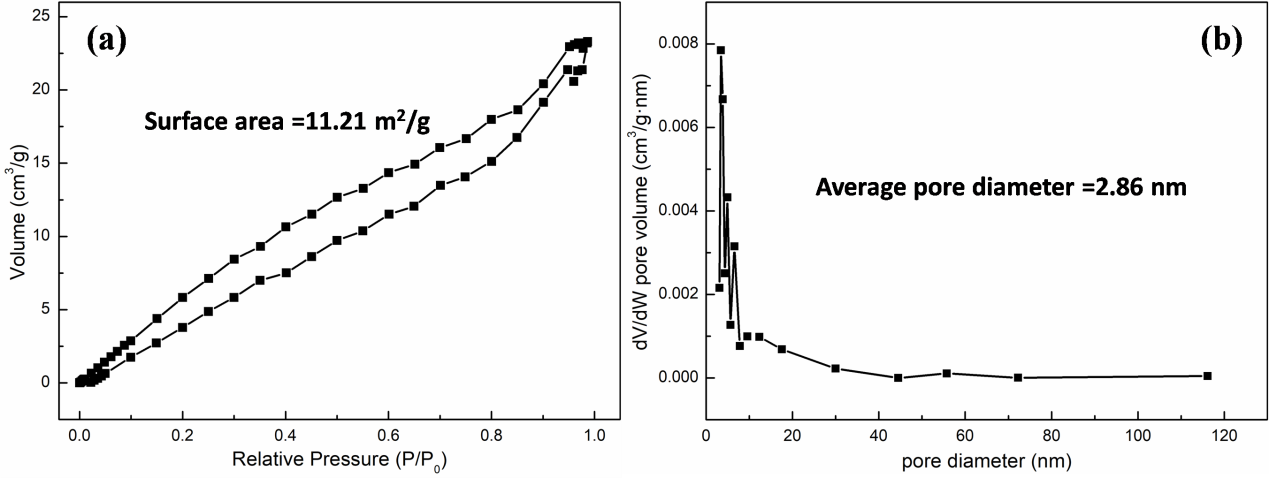


**Fig. S9** (a) The nitrogen adsorption-desorption isotherms and (b) pore diameter distribution of Ag/TiO_2_/PDA-bamboo.





**Fig. S10** Reusability test of Ag/TiO_2_/PDA-bamboo after 5-fold continuous decolorization cycles.





**Fig. S11** MB photodegradation ability of Ag/TiO_2_/PDA-bamboo and Ag/TiO_2_ catalysts.

**

**

**Fig. S12** Kinetic linear simulation curves of MB degradation with different samples


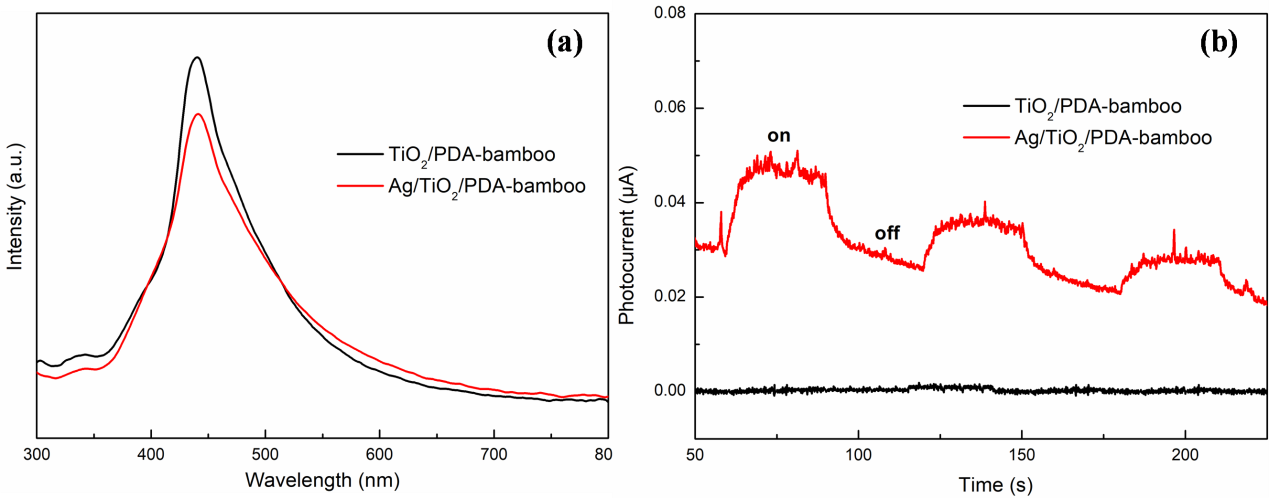


**Fig. S13** (a) PL spectra and (b) Transient photocurrent responses of Ag/TiO_2_/PDA-bamboo and TiO_2_/PDA-bamboo.





**Fig. S14** Trapping experiment of active species.


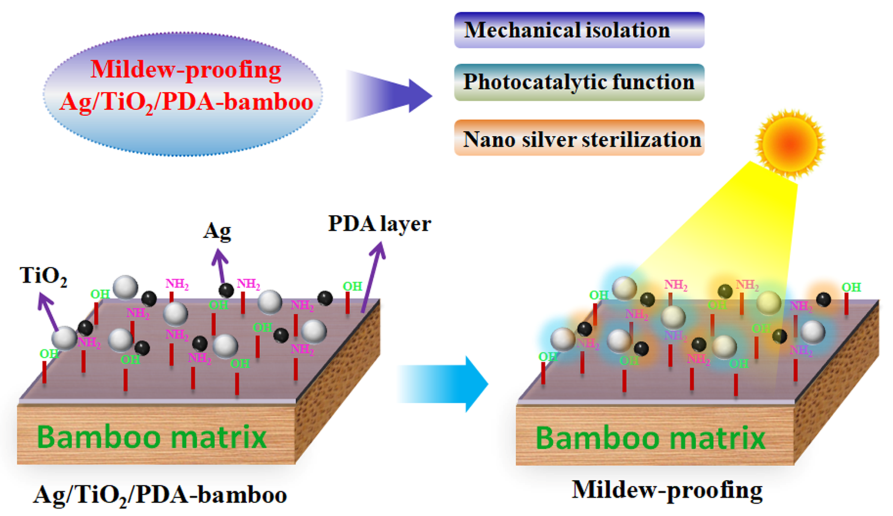


**Fig. 15** Schematic diagram illustrating a possible mechanism of the improved mildew-proofing capability of Ag/TiO_2_/PDA-bamboo.
